# Characterization of Extracts of Coffee Leaves (*Coffea arabica* L.) by Spectroscopic and Chromatographic/Spectrometric Techniques

**DOI:** 10.3390/foods11162495

**Published:** 2022-08-18

**Authors:** Lorenzo Cangeloni, Claudia Bonechi, Gemma Leone, Marco Consumi, Marco Andreassi, Agnese Magnani, Claudio Rossi, Gabriella Tamasi

**Affiliations:** 1 Department of Biotechnology, Chemistry and Pharmacy, University of Siena, Via A. Moro 2, 53100 Siena, Italy; 2 Centre for Colloid and Surface Science (CSGI), Via della Lastruccia 3, 50019 Sesto Fiorentino, Italy; 3 National Interuniversity Consortium of Materials Science and Technology (INSTM), Via G. Giusti 9, 50121 Firenze, Italy

**Keywords:** *Coffea arabica* L. leaves, secondary metabolites, bioactive compounds, xanthones, chlorogenic acids

## Abstract

*Coffea arabica* L. leaves represent a viable alternative to the canonical matrices used for preparation of beverages, such as tea leaves and grounded coffee beans. Coffee leaves infusions are rich in antioxidant phenolic compounds and have a lower concentration of caffeine. Due to increasing interest in this field, a complete study of the bioactive compounds as chlorogenic acids, xanthones and alkaloids is noteworthy. *C. arabica* leaves were subjected to ultrasound-assisted extraction, and the extracts were studied via nuclear magnetic resonance spectroscopy (NMR) and chromatographic techniques coupled with mass spectrometry (HPLC-MS^n^) to identify and quantify the secondary metabolites profile through an untargeted data dependent approach. A quantitative analysis was performed for the major components—chlorogenic acids, mangiferin, caffeine and trigonelline—via HPLC-MS in Single Ion Monitoring (SIM) mode. In total, 39 compounds were identified. The presence of these bioactive compounds proved the strong potential of *C. arabica* leaves as functional food and as an alternative to classic infused beverages.

## 1. Introduction

The *Coffea arabica* L. is the most prestigious species of the *Rubiaceae*, perennial plants from whose fruit, the coffee beans are obtained. The family includes several genus and species, but the greatest production of coffee is from *C. arabica* beans, which represent the most diffused variety, at 59%. South America has been, for the last hundred years and still today, the main production area. Currently, the American continent accounts for more than 50% of the world coffee production, with Brazil, Honduras, Mexico, Peru and especially Colombia among the major contributors to the world supply of coffee. During the last years, there has been a continuous and progressive interest increase in coffee leaves applications as a potential alternative to tea, and as food supplement [1]. Coffee leaves contain several phytochemical molecules such as alkaloids, flavonoids, terpenes, tannins, xanthonoids, phenolic acids, phytosterol, amino acids and carotenoids, which help to give coffee its antioxidant, anti-inflammatory, antihypertensive, anticancer, antibacterial and antifungal properties [2,3]. Furthermore, mangiferin has been previously investigated in relation to heart diseases. In particular, natural bioactive molecules have been studied as possible alternatives to synthetic drugs as potential inhibitors of the processes of activation and platelet aggregation [4,5].

The phytochemical profile of plants varies according to the cultivar, the growth region, the climate and the vegetation stage of the plants, together with the agronomical processes. Several studies have been already published in this field for vegetable species [6] and for coffee leaves [7]. The post-harvesting processes also affect the overall profile of bioactive molecules in the vegetable [8]. As regards coffee beans, they commonly undergo fermentation and roasting procedures to finally obtain the commercial ground coffee powder, that is usually used to make coffee beverages. These processes cause the degradation of most of the chlorogenic acids and other bioactive species that are initially present in the fruits [9]. On the contrary, the simpler drying procedures to obtain commercial coffee leaves, can allow the preservation of these bioactive molecules. In addition, the mangiferin, another powerful antioxidant molecule belong the xanthones family, has been identified and quantified in *C. arabica* leaves. Its presence in the pulp and peels of coffee berries was previously revealed [10], but it was never detected before in coffee leaves. This suggests that coffee leaves have a potentially beneficial profile that is much more important than the most well-known beans. Furthermore, the assessment of the antioxidant and other bioactive properties of the identified and quantified molecules is a very important topic already studied and reported in many papers [11,12,13]. Considering the promising beneficial effects on human health and the growing interest in the applications of coffee leaves, it is necessary to fully understand the profile of bioactive components. An important feature of coffee leaf infusions is related to the low concentration of caffeine with respect to regular tea leaves infusion beverages [14,15].This feature does not affect the polyphenolic concentration and other beneficial bioactive compounds. An infusion of coffee leaves constitutes a potentially healthier option to both coffee and tea. Coffee leaves are rich in phenolic compounds such as mangiferin and the esters of hydroxycinnamic acids (HCEs), which are not present in tea or coffee [1].

The present study aims to characterize coffee leaf extracts from *C. arabica* L. cultivar, Castillo variety to increase knowledge on antioxidant compounds present in coffee leaves using untargeted high throughput techniques such as tandem mass spectrometry coupled with high performance liquid chromatography (HPLC-MS^n^) and nuclear magnetic resonance spectroscopy (NMR). This experimental design has previously been applied for the study of different plant matrices such as *Olea europaea* L. and *Solanum lycopersicum* L. [16,17]. The novelty of this paper focuses on the optimization of a combined approach, of the two main spectroscopic and spectrometric techniques commonly processed for untargeted metabolomic studies, allowing high throughput. This is intended to avoid, as much as possible, potential critical biases in secondary metabolite identification that are often present in low concentrations, and to characterize the vegetable matrices in their natural complexity. After identification, the main categories of compounds were quantified to assess the concentration of bioactive compounds in *C. arabica* L. leaves.

## 2. Materials and Methods

### 2.1. Chemicals and Reagents

All the reagents and solvents listed below were purchased from Sigma-Aldrich (Milan, Italy) and were used without any further purification: mangiferin (≥98.0%), sinapic acid (≥98.0%), quinic acid (≥96.0%), 3,5-dicaffeoylquinic acid (≥95.0%), chlorogenic acid (5 CGA, ≥95.0%), neo-chlorogenic acid (3-CGA, ≥98.0%), cripto-chlorogenic acid (4-CGA, ≥98.0%), quercetin (≥95.0%), quercetin-3β-(D)-glucoside (≥98.0%), trigonelline hydrochloride (≥95.0%), nicotinic acid (≥99.5%), theophylline (≥98.0%), methanol (MeOH, LC-MS grade 99.9%), ethanol (EtOH, LC-MS grade 99.9%), acetonitrile (CH_3_CN, ACN, LC-MS grade), formic acid (HCOOH, LC-MS grade 98.5%), deuterated methanol (CD_3_OD, MeOD-d_4_; 99.8 atom % D). Caffeine (≥98%) was purchased from Extrasynthese (Lione, France). Ultrapure water (18.2 MΩ·cm) was obtained from a Rephile Direct-Pure water purifier.

### 2.2. Plant Material Collection, Pre-Treatment and Extraction

The *Coffea arabica* L. leaves were collected in Colombia, in the Department of Huila at Garzon, located in the central part of the country at an altitude of about 1700 m asl, on a cultivation of Castillo variety. Once collected, the coffee leaves underwent a freeze-drying procedure, to allow the conservation of the bioactive components over time. The leaves were frozen under liquid nitrogen and then freeze-dried (–48 ± 2 °C, 450 ± 50 μBar) for 96 h. After the freeze drying process, the samples were cold crushed in a knife mill using liquid nitrogen (Pulverisette 11, Fritsch) and sieved to obtain a particle size <500 μm. The ground leaves were stored at –20 ± 1 °C before subsequent analyses.

The extraction protocol was optimized on the basis of procedures previously reported, with some modifications [8,18]. Aliquots of 0.500 g of lyophilized sample (analytically weighed) were treated with 10 mL of a solvent mixture consisting of EtOH/H_2_O (70:30, *v*/*v*). The extraction process was assisted by ultrasound sonication using an ultrasonic bath (10 min, 20 ± 1 °C; nominal power 120 W; ultrasound frequency 35 kHz; Branson Ultrasonics Corporation, Danbury, CT, USA). The suspension was then centrifuged (5 min, 1882 g; Thermo Electron Corporation PK 110 centrifuge). The surnatant was carefully separated from the solid residue, and then transferred into a polypropylene tube. The extraction procedure was repeated three times, using 10 mL of mixture (each time) on residual solid phase. The aliquots of extracts were combined (total volume, 30 mL) and the extract was dried overnight under a gentle nitrogen flow, and finally stored in polyethylene tubes at –20 ± 1 °C before subsequent analyses.

### 2.3. Nuclear Magnetic Resonance (NMR) Experiments

NMR experiments were performed using a Bruker DRX-600 Avance spectrometer operating at 600.13 MHz for ^1^H, equipped with an xyz gradient unit. Spectra were processed using Bruker TopSpin software (version 3.6.1, Bruker, Bremen, Germany). The dried extract was freeze dried to remove the eventual humidity, and then reconstituted in deuterated methanol (MeOD-d_4_) for NMR analysis.

### 2.4. Chromatographic Conditions

The coffee leaves extract was resuspended in MeOH/H_2_O mixture (40:60 *v*/*v*) and the analyses were performed using an HPLC instrument (Thermo Fisher Scientific UltiMate 3000) coupled to a linear ion trap mass spectrometer (Thermo Fisher Scientific LTQ XL), equipped with an electrospray ion source (ESI). The spectra were acquired and processed using Xcalibur software (Thermo Fisher Scientific, Waltham, MA, USA). A biphenyl column was used for the analysis (Biphenyl Phenomenex Kinetex, 100 × 2.1 mm; particle diameter 2.6 μm; porosity 100 Å) with a phenyl Security Guard pre-column (4.0 × 2.0 mm, Phenomenex, Torrance, CA, USA). The column temperature was 35 ± 1 °C. The eluents were (A) H_2_O and (B) MeOH, both acidified with formic acid (0.1%), and the gradient of elution was optimized based on z subsequent positive or negative ESI process, as reported hereafter. Gradient for ESI negative mode: from 0.0 to 15.0 min 10–15% B (linear); from 15.0 to 25.0 min 15–50% B (linear); 25.0 to 35.0 min 50–95% B (linear). Gradient for ESI positive mode: from 0.0 to 5.0 min 0% B (isocratic); from 5.0 to 45.0 min 0–70% B (linear). In both cases, the injected volumes were 3 μL, and the flow rate was 0.4 mL/min. Each standard and sample was injected and analyzed in triplicate.

### 2.5. HPLC-MS^n^ Methodology for Qualitative and Quantitative Analysis of the Extracts

To identify the analytes in the extracts, the MS^n^ spectra products from the sequentially fragmented molecules within the ion trap were compared with the spectra of the characteristic fragments obtained from the standards and spectra reported in literature. The main feature of this method is to take advantage of the linear ion trap ability to fragment a specific ion inside the trap, separating it in time, unlike what happens in other analyzers that separate in space (Q, TOF, EB).

To characterize the extracts, a preliminary approach was used to collect information on the structure and the fragmentation of the various analytes by using a data dependent acquisition approach. Each MS scan in both positive and negative modes above a specific threshold level was collected and the most abundant ion was fragmented through CID (collision induced dissociation) using He as a collision gas, generating a MS^2^ spectra; the first and second most abundant ions were then fragmented again in two separate steps, generating two different MS^3^ spectra. The generated spectral fragmentation tree was used to preliminarily identify the constituents of the extracts. All the identified constituents were compared with spectral databases (MassBank, mzcloud, HMDB) and the relative standard compounds when available. The MS full scan analysis were in the range of *m*/*z* 100–1000. The collision energies were 35 nCE (normalized collision energy) and 45 nCE respectively for MS^2^ and MS^3^ fragmentation steps.

The ESI parameters were optimized in both positive and negative modes through direct injection of the standards of the major components of the extracts: pure caffeine, chlorogenic acid, mangiferin and rutin, dissolved in a MeOH/H_2_O (60:40, *v*/*v*) mixture. For negative ionization, the following parameters were optimized: spray voltage 3000 V, sheath gas and auxiliary gas pressure of 35 and 25 arbitrary units, respectively, capillary temperature 350 °C. For positive ionization, the following parameters were optimized: spray voltage 3500 V, sheath gas and auxiliary gas pressure of 20 and 12 arbitrary units, respectively, capillary temperature 300 °C.

The single ion monitoring (SIM) method was used for the quantitation of all caffeoylquinic derivatives, mangiferin and alkaloids, selecting ions with the values of *m*/*z*, as shown in Table 1. All molecules except alkaloids were quantified in negative mode via external calibration method using the external calibration method with internal standards. The calibration curves of the analytes were acquired in triplicate and obtained by plotting the area ratio of the analytes normalized by the internal standard against the analyte concentration in the linearity ranges (Table 1). Chlorogenic acids were quantified using the calibration curve built from 5-caffeoylquinic acid, while mangiferin isomers were quantified on the curve obtained from a standard of mangiferin. For the quantitation of dicaffeoylquinic acids, a calibration curve obtained from 3,5 dicaffeoylquinic acid was used. For the alkaloids, each molecule was quantified on its relative standard. The results were expressed as g/kg of sample dry weight (DW).

## 3. Results and Discussion

### 3.1. ^1^H NMR Analysis of Coffea Arabica Leaves Extract

**Figure 1:**
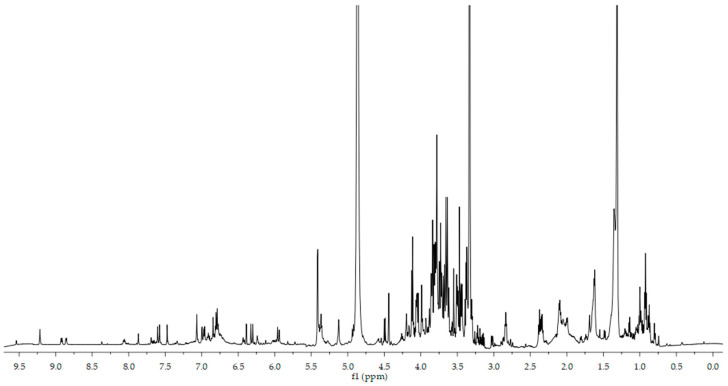
^1^H NMR (600 MHz) spectrum of *Coffea arabica* L. leaf extract resuspended in deuterated methanol.

NMR spectroscopy was used to characterize the main components of the extract without any further purification. The acquired ^1^H spectrum for the coffee leaf extract reconstituted in CD_3_OD is reported in Figure 1. The signals reported in the spectrum were analyzed and compared with the available standards in already published papers [19,20] and databases [21,22] (HMDB, BMRB). The spectrum can be divided into three main sections based upon the chemical shift values of the principal compounds.

**Figure 2:**
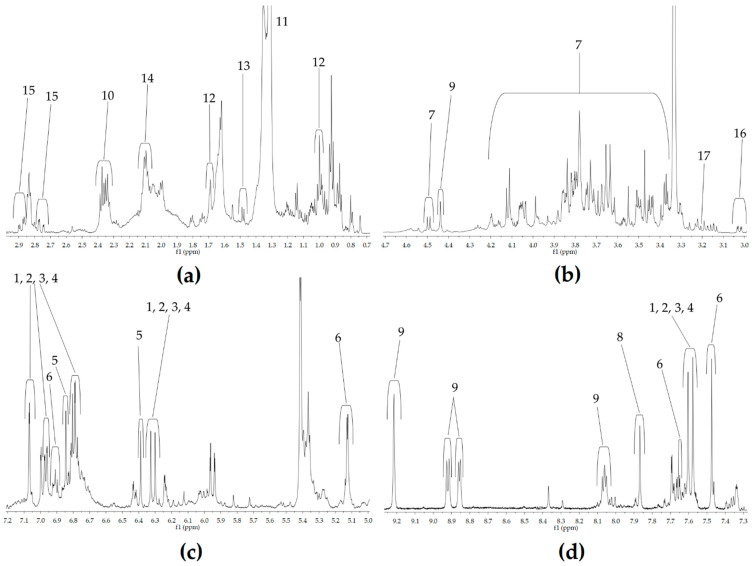
Magnification of the four main section of the ^1^H NMR spectrum of *C. arabica* L. leaf extract: (**a**) 0.0–3.0 ppm, (**b**) 3.0–5.0 ppm, (**c**) 5.0–7.2 ppm, (**d**) 7.3–9.3 ppm.

The assignment of the signals is reported in Figure 2 and the chemical shift values are reported in Table 2, together with the multiplicities and coupling constants.

The first section (0.0–3.0 ppm, Figure 2a) corresponds to the region usually associated with aliphatic compounds, amino acids and organic acids [20]. In this part of the spectrum are located the signals of compounds **12**–**17**, respectively assigned to malic and lactic acid, Leu, Ala, Glu, and Asp. The most intense signals are located in the central part of the spectrum, from 3.0 to 5.0 ppm (Figure 2b): This section is dominated by carbohydrates signals, principally attributed to hexose, deoxyhexose and pentose monosaccharides involved in the glycosylation of the main compounds. In addition, Cys (**18**) and choline (**19**) are assigned in this interval at chemical shift values of 3.0 and 3.2 ppm, respectively. The downfield section (5.0–10.0 ppm; Figure 2c,d) contains signals that arise from aromatic molecules; the main components identified are phenolic compounds as chlorogenic acids (compounds **1**, **2**, **3**, **4**), caffeic acid (**5**), the flavonoid rutin (**7**) and the xanthone mangiferin (**6**). Another important class of compounds identified in this spectral section correspond to alkaloids, specifically caffeine (**10**) and trigonelline (**11**), located in the most downfield section of the spectrum (7.0–9.5 ppm, Figure 2d).

### 3.2. HPLC-ESI(−)-MS^n^ Profiling of Coffea Arabica Leaf Extract

**Figure 3:**
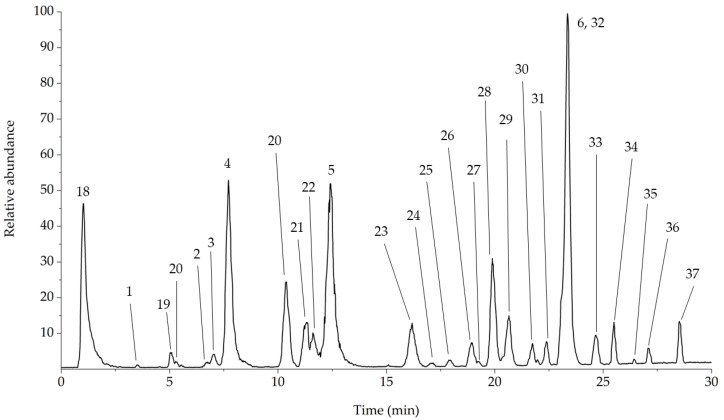
HPLC-ESI(−) chromatogram of coffee leaves extract. The identified compounds are numbered as also reported Table 3.

The dried extracts were resuspended in MeOH/H_2_O (60:40, *v*/*v*) filtered through a 0.2 μm PTFE syringe filter (Whatman) before injection, and analyzed through data dependent analysis, resulting in the chromatogram reported in Figure 3.

The identified compounds are members of different categories of phenolic compounds—xanthones, flavonoids, chlorogenic acids, and lignans—except the first compound (**18**) with *m*/*z* 191, which was not retained in the chromatographic elution,. After accurate analysis of the product ions generated from MS^2^ fragmentation shown in Table 3, the compound was identified as quinic acid; this was confirmed through comparison with the available standard.

#### 3.2.1. Xanthones

**Figure 4:**
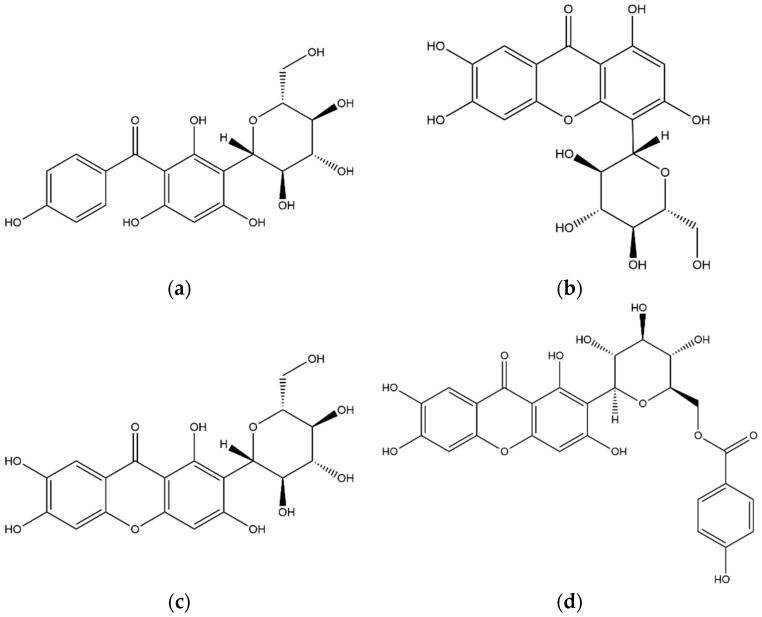
Structures of selected xanthones identified in the coffee leaf extract: (**a**) iriflophenone 3-C-glucoside, (**b**) isomangiferin, (**c**) mangiferin, (**d**) 6-O-(*p*-hydroxybenzoyl)mangiferin.

The first compound related to the xanthone structure was compound **19**. The deprotonated molecule shows a [M−H]^−^ ion at *m*/*z* 407, producing ions at *m*/*z* 287 and 317 in MS^2^ and 193, 243, 167 in MS^3^ fragmentations. The compound was identified as iriflophenone-3-C-glucoside (Figure 4a), in agreement with database spectra and previous published papers [23]. Other xanthone molecules were eluted at Rt of 11.63 and 12.42 min, respectively; the molecules show the same MS^n^ fragmentation with a [M−H]^−^ ion at *m*/*z* 421, and were identified as isomangiferin (**22,** Figure 4b) and mangiferin (**5,** Figure 4c). The same fragmentation pattern of the two molecules required the optimization of the chromatographic separation, and the consequent structure assignment was based on comparison with Rt and MS^n^ fragments of the standard mangiferin. The last compound identified as xanthone was eluted at Rt 27.10 min; the deprotonated molecule shows *m*/*z* 541 and the most abundant ions in MS^2^ spectrum were at *m*/*z* 331 and 301, as reported for compounds **22** and **5**. The ion at *m*/*z* 331 was consequently fragmented, generating product ions at *m*/*z* 313, 301, 271 and 259. These fragmentations led to the identification of compound **37** as 6-O-(*p*-hydroxybenzoyl) mangiferin (Figure 4d), in agreement with database spectra and previous published papers [24].

#### 3.2.2. Chlorogenic Acids

**Figure 5:**
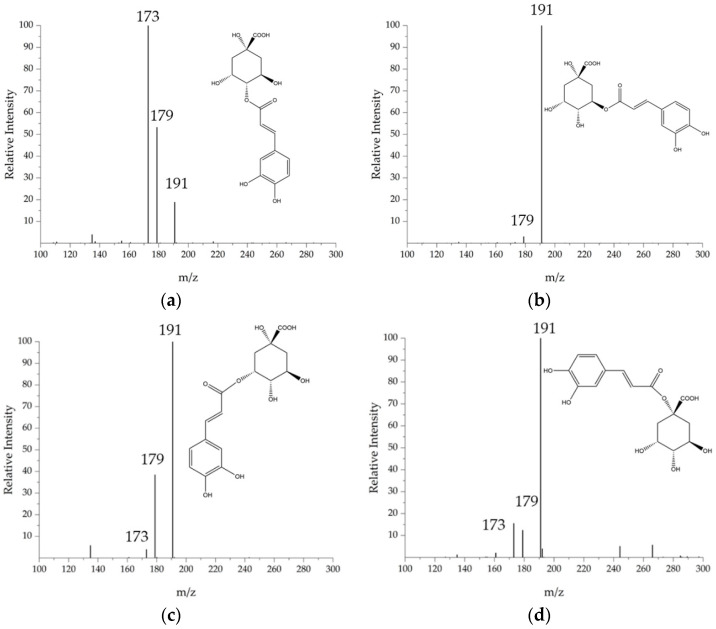
Tandem mass spectra of the compounds **1**, **2**, **3**, and **4**, identified as chlorogenic acids: (**a**) 4-CGA, (**b**) 5-CGA, (**c**) 3-CGA, (**d**) 1-CGA.

This class of molecules is represented by 5-caffeoyl-quinic acids and is composed by esters of hydroxycinnamic acids (ferulic, caffeic, *p*-coumaric acids) with quinic acid. They are widely studied [25,26,27,28,29] for their antioxidant properties and high concentrations in plant matrices. Mono caffeoyl-quinic acids have [M−H]^−^ ions at *m*/*z* 353 and their differentiation is based upon the MS^2^ fragmentation as reported from literature [25]. In this study, four different species were isolated at Rt values, as reported in Table 3; the species identified are 3-CGA (**1**), 1-CGA (**2**), 4-CGA (**3**) and 5-CGA (**4**). The structural assignment was based on the comparison with the relative standards for compounds **1**, **3** and **4**, while for compound **2,** the tentative structural identification was based on literature [25]. The MS^2^ fragmentation of mono caffeoyl-quinic acids is reported in Figure 5.

**Figure 6:**
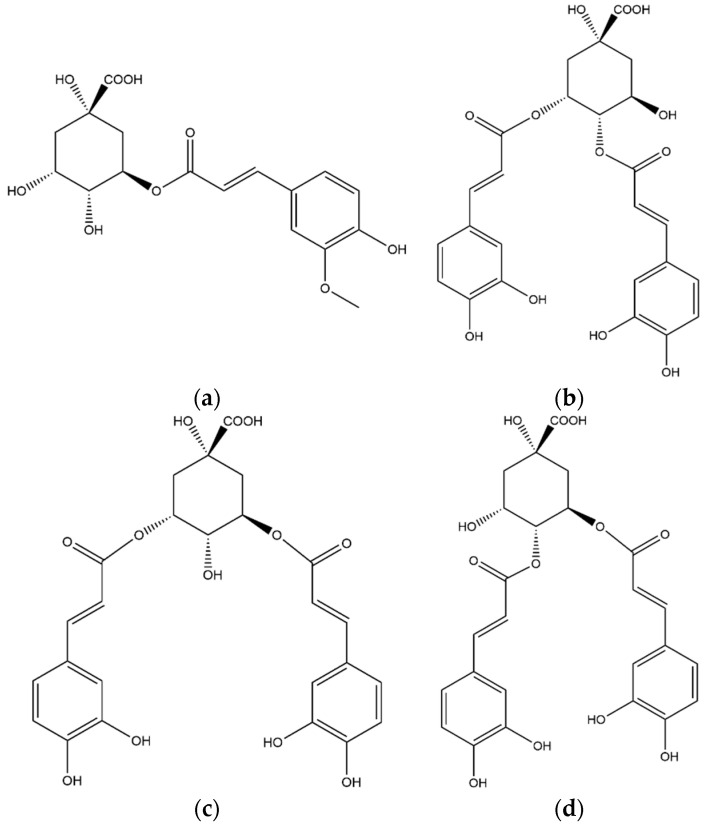
Structures of the chlorogenic acids identified in the coffee leaf extract: (**a**) 5-feruloyl-quinic acid, (**b**) 3,4-dicaffeoyl-quinic acid, (**c**) 3,5-dicaffeoyl-quinic acid, (**d**) 4,5-dicaffeoyl-quinic acid.

Compound **28** was eluted at Rt 19.25 min with a [M−H]^−^ ion at *m*/*z* 367; after the first fragmentation, the MS^2^full scan spectrum presented two main fragments at *m*/*z* values of 191 and 16; the following fragmentation of the *m*/*z* 191 ion produced three fragment ions at *m*/*z* values 127, 173 and 171. The compound **28** was identified as 5-feruloyl-quinic acid (5-FQA, Figure 6a) by comparison with data previously reported [25].

The dicaffeoyl-quinic acids are represented by the ion [M−H]^−^ at *m*/*z* 515; in the present study three compounds with the same *m*/*z* value were eluted at Rt 24.00 (**33**), 24.62 (**34**), and 26.44 (**36**) min, respectively. The three compounds were identified, as reported in literature [17], as 3,4-dicaffeoylquinic acid (**33,** Figure 6b), 3,5-dicaffeoylquinic acid (**34,** Figure 6c) and 4,5-dicaffeoylquinic acid (**36,** Figure 6d).

#### 3.2.3. Flavonoids

**Figure 7:**
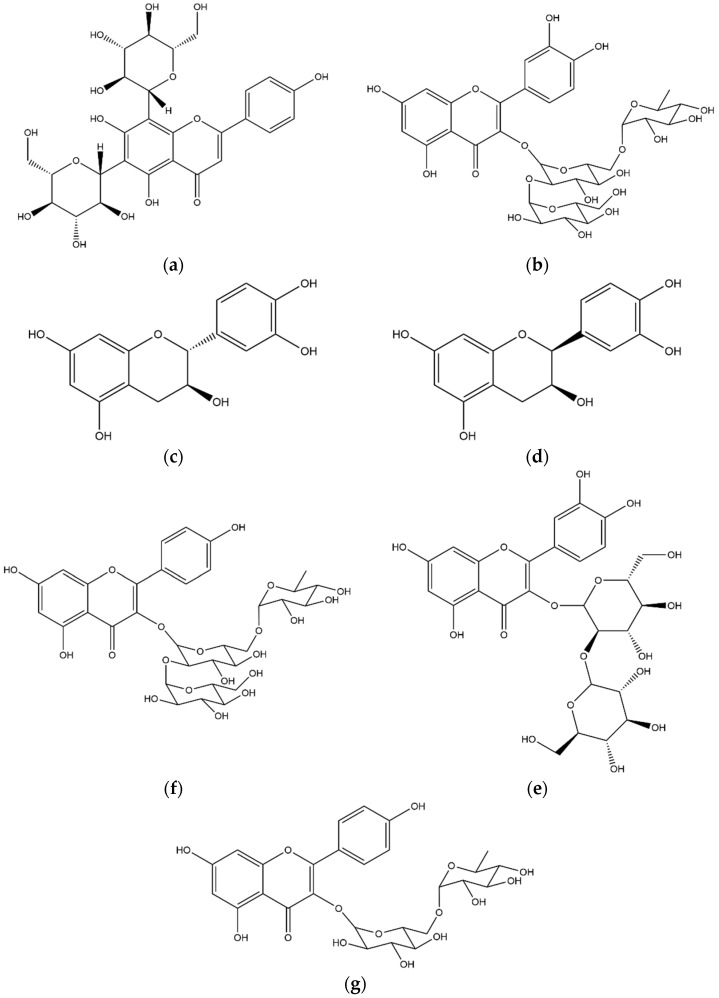
Structures of the main flavonoids identified in the coffee leaf extract: (**a**) apigenin 6,8-di-C-glucoside, (**b**) rutin glicoside, (**c**) (+)catechin, (**d**) (+)epicatechin, (**e**) quercetin sophoroside, (**f**) kaempferol triglycoside, (**g**) kaempferol rhamnoglucoside.

Flavonoids are a class of phenolic molecules widely distributed in plant matrices that have important antioxidant activities [29]. The compound **26** was eluted at 17.91 min with a [M−H]^−^ ion at *m*/*z* 593. The fragmentation of the deprotonated molecule produced a MS^2^ spectra with the base peak at *m*/*z* 473, together with the ions at *m*/*z* 503, 383 and 353. The main neutral loss of 120 Da was addressed as a loss of C-glucoside moiety. This fragmentation pattern, connected with the absence of the regular neutral loss of 162 Da previously observed for O-glycosides [30,31], allowed the identification of the compound as a C-glucoside derivative. The *m*/*z* 473 ion was then fragmented, producing an MS^3^ spectrum with a base peak at *m*/*z* 353, confirming a consecutive loss of C-glucoside moiety. The comparison of these fragmentation patterns with data previously published [32] and with databases (mzcloud) allowed the identification of compound **26** as apigenin 6,8-di-C-glucoside (Figure 7a).

The second flavonoid was eluted at Rt 19.89 min (compound **29**) with a base peak ion at *m*/*z* 771. The fragmentation produced a MS^2^ spectra reporting the classic main losses of O-glycosylated flavonoids, producing the ions at *m*/*z* 609 ([M−Hexose−H]^−^), 591 ([M−Hexose−H_2_O−H]^−^) and 301 ([M−2Hexose−Deoxyhexose−H]^−^). The MS^3^ fragmentation of the MS^2^ base peak at *m*/*z* 591 produced several fragments consistent with the identification of a glycosilated rutin molecule (quercetin rutinoside). The identification of compound **29** as rutin glycoside (Figure 7b) was also suggested [33].

Two compounds with the same [M−H]^−^ ion at *m*/*z* 289 were eluted at Rt 5.28 min and 10.38 min. The two compounds show the same fragmentation pattern in both MS^2^ and MS^3^ fragmentation steps. The fragmentation was compatible with the one obtained for the catechin and epicatechin standards, and was compared with databases and literature [34]. Compounds **20a** and **20b** were identified as catechin/epicatechin (Figure 7c,d).

Compound **30** showed a Rt 20.70 min and a base peak ion at *m*/*z* 625; the compound was identified as quercetin sophoroside (Figure 7e) based on the relative abundances and the values of the neutral losses in MS^2^ and MS^3^ spectra. In MS^2^, four main product ions were identified as [M−Hexose−H]^−^ (*m*/*z* 463), [M−Hexose−H_2_O−H]^−^ (*m*/*z* 445) and [M−2Hexose−H]^−^ (*m*/*z* 301). The MS^3^ fragmentation was performed on the ion at *m*/*z* 301 and corresponded to the ESI negative fragmentation of the quercetin aglycone. The identification of compound **30** was also confirmed by NMR and tandem mass spectrometry data in literature [33,35].

Kaempferol triglycoside was eluted at Rt 22.41 min and reported in Table 3 as compound **32 (**Figure 7f). The MS^2^ tandem mass spectrum evidenced two main fragments at *m*/*z* 575, identified as [M−Hexose−H_2_O−H]^−^ ion and 285 ([M−3Hexose−H]^−^), that showed a MS^3^ fragmentation pattern compatible with the kaempferol aglycon. This compound had already been already identified in *Coffea arabica* leaves and identified through NMR analysis [33]. The most intense peak in the ESI negative chromatogram was identified as compound **6** (rutin, quercetin rutinoside); the fragmentation of this widely distributed flavonoid is largely described in literature. The most intense fragments are related to the loss of the rutinose moiety (Glu-Rha), producing the aglycon fragment at *m*/*z* 301 ([M−Glu−Rha−H]^−^). The *m*/*z* 301 base peak showed a consequent fragmentation consistent with the quercetin aglycon reported in literature [36].

The last flavonoid eluted was compound **35** with a Rt of 25.50 min and a [M−H]^−^ ion at *m*/*z* 593 (Figure 7g), generating a MS^2^ fragmentation spectrum with the only presence of the ion at *m*/*z* 295; the consequent MS^3^ fragmentation produced a spectrum compatible with the kaempferol aglycon. The absence of intermediate neutral losses suggested the identification of the compound **35** as kaempferol rhamnoglucoside [37].

#### 3.2.4. Lignans

**Figure 8:**
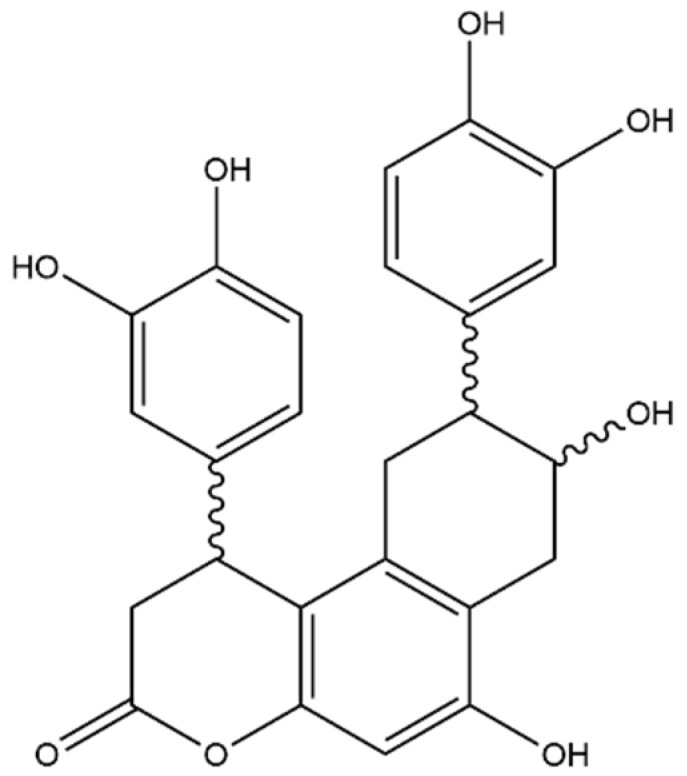
Structure of the identified lignans (compounds **31a** and **31b**) cinchonain I isomers.

Lignans are spread throughout the plant kingdom and high concentrations of these compounds have been measured in various matrices [38]. Compounds **31a** and **31b** were eluted at Rt 21.75 and 28.52 min, respectively; both compounds show a [M−H]^−^ ion at *m*/*z* 451. The fragments generated both in MS^2^ and MS^3^ fragmentation are identical, suggesting that the two compounds are isomers of the same molecule. The MS^2^ fragmentation showed only one fragment at *m*/*z* 341; this fragment is generated from the loss of a catechol unit with a neutral loss of 110 Da. The following fragmentation of the *m*/*z* 341 produced three main fragments at *m*/*z* 231, 219 and 217, respectively. The ion at *m*/*z* 231 was generated by a second neutral loss of catechol (110 Da). Comparing the tandem mass spectrometry information with data previously published [39,40,41], the compounds **31a** and **31b** were identified as cinchonain I isomers (Figure 8).

#### 3.2.5. Procyanidins

**Figure 9:**
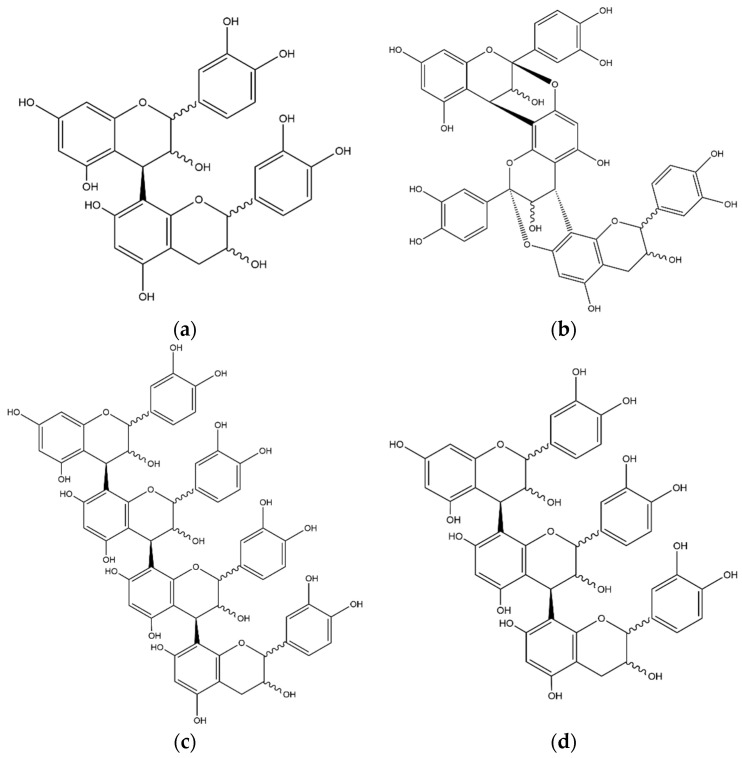
Structure of the identified procyanidins: (**a**) procyanidin B, (**b**) procyanidin dimer A-type, (**c**) procyanidin tetramer B-type, (**d**) procyanidin C.

Procyanidins are polyphenols abundant in a wide distribution of plant species with a multitude of chemo-preventive bioactive effects. The compound **21** eluted at Rt 11.22 min was identified as procyanidin B showing a [M−H]^−^ ion at *m*/*z* 577 (Figure 9a). The molecule was subjected to CID (collision induced dissociation) to obtain the fragments at *m*/*z* 425, 407 and 289. The 152 Da neutral loss that generated the fragment at *m*/*z* 425 is generated from a retro-Dies–Alder fragmentation that is typical of procyanidins, while the ion at *m*/*z* 289 is generated from the cleavage of the covalent bond between the two monomeric units of procyanidins [42,43]. Compound **21** was then identified through comparison with databases and Rt and fragmentation of the relative standard.

The following elution at Rt 16.19 was attributed to the deprotonated molecule with *m*/*z* 863, corresponding to compound **24**. This compound shown a fragmentation pattern similar to compound **21** and was characterized from the neutral loss of 152, typical of the procyanidins molecules, consisting in a retro Dies–Alder fragmentation mechanism. Comparing the fragmentation of this compound with the literature [44] and databases, compound **24** was identified as procyanidin trimer A-type (Figure 9b).

Compound **25** was eluted at Rt 17.10 with a base peak at *m*/*z* 576; this compound was fragmented, producing the most abundant fragment at *m*/*z* 550 (neutral loss 76 Da), together with the ions at *m*/*z* 491, 289 and 451. The low intensity of the MS^2^ signal did not allow MS^3^ fragmentation, but the peculiar loss of 76 Da was used to tentatively identify compound **25** as a procyanidin tetramer doubly charged ion [M−2H]^2−^ (Figure 9c), as reported in literature [45].

The procyanidin eluted at Rt 18.95 produced a [M−H]^−^ ion at *m*/*z* 865 and generated MS^2^ fragments at *m*/*z* 739, 695 and 577, and the following fragmentation of the MS^2^ base peak (*m*/*z* 695) produced fragments at *m*/*z* 543, 451, 405 and 289. The fragments of MS^3^ are comparable with smaller procyanidins showing the catechin/epicatechin monomer at *m*/*z* 289. The comparison of the fragmentation pattern allowed the identification of compound **27** as procyanidin C (Figure 9d), as reported in literature [46].

### 3.3. HPLC-ESI(+)-MSn Profiling of Coffea Arabica Leaf Extract

**Figure 10:**
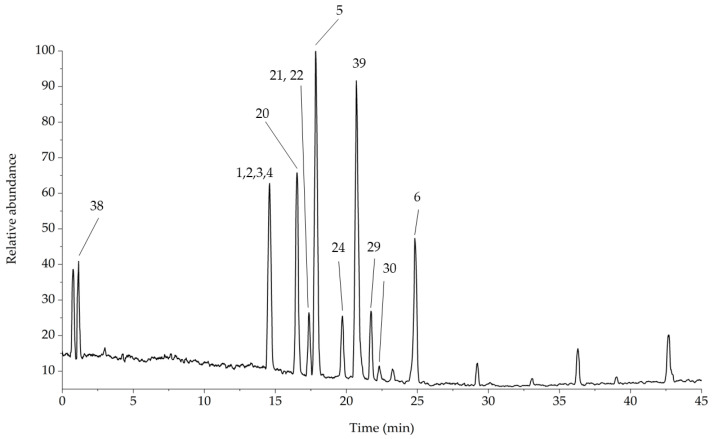
HPLC-ESI(+) chromatogram of coffee leaves extract. The identified compounds are numbered as also reported Table 3.

The acquisition of the positive ionization chromatogram was necessary to study different classes of compounds contained in the extract, together with the same species already characterized in the previous paragraph. The obtained chromatogram is reported in Figure 10.

**Figure 11:**
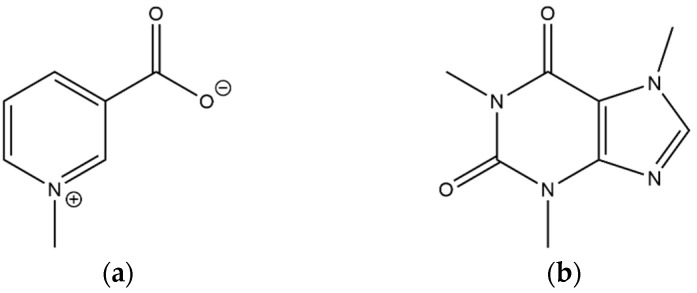
Structure of the identified alkaloids: (**a**) trigonelline, (**b**) caffeine.

In Figure 10, 14 compounds are identified, 12 of which were already identified through the analysis of the ESI negative ionization chromatogram. Only compounds **38** and **39** were absent in the previous chromatogram and were classified as alkaloids. The high noise visible in the baseline is caused from the 100% aqueous mobile phase composition used in the first section of the chromatographic gradient; the use of fully aqueous mobile phases has a deleterious effect on ionization yield and ESI spray stability, but was necessary to load the highly polar compound **38** on the column stationary phase. Compound **38** showed a very short Rt of 1.11 min, emphasizing the very high polarity of this molecule. The protonated molecule shows a [M+H]^+^ ion at *m*/*z* 138. The fragmentation of the *m*/*z* 138 ion generated only one fragment at *m*/*z* 121 with a mass shift of 17 Da, suggesting an ammonia loss. Comparing the Rt values and the fragmentation with the relative standard, compound **38** was identified as trigonelline (Figure 11a). The second alkaloid (compound **39**) was identified as caffeine; the presence of this alkaloid in coffee leaves is very well known in common knowledge. The fragmentation of caffein produced the MS^2^ ion at *m*/*z* 138 and the following MS^3^ product ion at *m*/*z* 121. The identification of compound **38** was performed through direct comparison with the relative standard (Figure 11b).

### 3.4. HPLC-MS Quantitation of Coffea arabica L. Leaves Extract Components

The main components of the *Coffea arabica* L. leaf extracts were quantified in both positive and negative mode to quantify mono-caffeoylquinic acids, mangiferin and isomangiferin, dicaffeoylquinic acids and alkaloids. The single ion monitoring (SIM) method was used for the quantitation of all caffeoylquinic derivatives, mangiferin and alkaloids, selecting ions with the values of *m*/*z* shown in Table 1. All molecules, except alkaloids, were quantified in negative mode.

Table 4 shows the quantitative values obtained for selected bioactive molecules in the *Coffea arabica* leaves extracts. Among the chlorogenic acids, the isomer esterified in position 5 (5-CGA) is the most abundant (16.27 ± 1.66 g/kg DW), while the other two isomers (3-CGA and 4-CGA) were found at lower concentrations, 8% and 0.2% of the 5-CGA content, respectively. As for dicaffeoquinic acids, the three isomers have comparable concentrations, with the 4,5-dCQA isomer having a concentration of 0.91 ± 0.05 g/kg DW, while the lower concentration was found for the 3,5-dCQA isomer (0.58 ± 0.02 g/kg DW). Mangiferin was revealed at a concentration of 4.43 ± 0.14 g/kg DW, while its isomer reveled a concentration about eight times lower (0.52 ± 0.03 g/kg DW). For caffeine and trigonelline, the measured amounts were 7.94 ± 0.42 and 4.47 ± 0.12 g/kg DW, respectively.

The concentration data resulted comparable to those already reported in literature, although the concentration of 5-CGA usually appears to be higher [7,47,48]. A suggested explanation could be related to the critical variability of many factors, such as fluctuations in environmental and pedoclimatic parameters and agronomic treatments.

## 4. Conclusions

A combined NMR and HPLC-MS approach was developed with the aim to separate, identify and quantify bioactive components in plant matrices belonging to the *Coffea arabica* L. family, Castillo variety. These molecules are important components of the pool of natural compounds responsible for the main beneficial effects of coffee leaves, as regards antioxidant activity. The results emphasize how coffee leaves represent an important source of bioactive compounds as functional foods. The combined use of two powerful techniques, chromatography coupled with tandem mass spectrometry and NMR spectroscopy, allowed a sound characterization of the secondary metabolites.

## Figures and Tables

**Table 1:** List of the quantified compounds in the *C. arabica* extracts with the relative internal standards and ESI ionization mode and calibration curve parameters.

No.	Compound	Internal Standard	ESI Mode	[M−H]^−^ [M+H]^+^	Equation	R^2^	Linearity Range (μg/mL)	LOD//LOQ (μg/mL)
**1**	3-CGA	Sinapic acid	(−)	353	y = 0.7405x	0.9986	0.500–10.0	0.07//0.30
**3**	4-CGA	Sinapic acid	(−)	353	y = 0.7405x	0.9986	0.500–10.0	0.07//0.30
**4**	5-CGA	Sinapic acid	(−)	353	y = 0.7405x	0.9986	0.500–10.0	0.07//0.30
**34**	3,5-dCQA	Sinapic acid	(−)	515	y = 0.3951x	0.9961	0.500–10.0	0.10//0.35
**33**	3,4-dCQA	Sinapic acid	(−)	515	y = 0.3951x	0.9961	0.500–10.0	0.10//0.35
**36**	4,5-dCQA	Sinapic acid	(−)	515	y = 0.3951x	0.9961	0.500–10.0	0.10//0.35
**5**	Mangiferin	Quercetin	(−)	421	y = 2.9624x	0.9995	0.025–2.50	0.007//0.023
**22**	Isomangiferin	Quercetin	(−)	421	y = 2.9624x	0.9995	0.025–2.50	0.007//0.023
**39**	Caffeine	Theophylline	(+)	138	y = 6.5298x	0.9997	0.025–12.5	0.004//0.010
**38**	Trigonelline	Nicotinic acid	(+)	195	y = 0.8923x	0.9983	0.100–12.5	0.02//0.06

**Table 2:** Assignment of ^1^H NMR signals in *C. arabica* L. leaf extracts.

No.	Compound	^1^H Chemical Shifts (ppm) [J-Coupling Values (Hz)]
**1, 2, 3, 4**	Chlorogenic acids	6.32 (d, 15.74), 6.80 (d, 8.14), 6.97 (dd, 1.9,1.9), 7.07 (d, 2.0), 7.59 (d, 15.9)
**5**	Mangiferin	6.39 (s), 6.84 (s)
**6**	Rutin	5.13 (d, 3.62), 7.65 (d, 2.4) 6.90 (m), 7.48 (s)
**7**	Saccharides	4.50 (d, 7.7), 3.3–4.2
**8**	Caffeine	7.87 (s)
**9**	Trigonelline	8.06 (t, 7.61), 9.22 (s), 8.86 (d, 5.9), 8.92 (d, 7.8), 4.47 (s)
**10**	Malic acid	2.37 (dd,15.3, 10.0)
**11**	Lactic acid	1.34 (d, 10.5)
**12**	Leucine	0.98 (t, 7.2), 1.69 (m)
**13**	Alanine	1.46 (d, 7.2)
**14**	Glutamine	2.12 (m)
**15**	Aspartic acid	2.75 (dd, 15.2, 8.3), 2.85 (dd, 17.3, 4.1)
**16**	Cystein	3.03 (dd, 2.7, 2.8)
**17**	Choline	3.22 (s)

**Table 3:** Assignment of identified compounds in HPLC-ESI chromatograms of coffee leaf extracts. The *m*/*z* values reported in bold represent the base peak of each spectrum. In MS^2^ spectra the base peak was fragmented to generate the MS^3^ fragmentation spectra. The MS^2^ and MS^3^ fragmentations are from HPLC-ESI chromatograms acquired in negative mode.

No.	Compound	Rt (min)	[M−H]^−^	[M+H]^+^	MS^2^	MS^3^
**18**	Quinic acid	1.01	191	-	127–**173**–111–93	-
**1**	3-Caffeoyl-quinic acid	3.51	353	355	179–**191**	**126**–173–171
**19**	Iriflophenone 3-C-glucoside	5.07	407	-	317–**287**	243–**193**–167
**20a**	Catechin/Epicatechin	5.28	289	291	271–**245**–205–179	227–**203**–161
**2**	1-Caffeoyl-quinic acid	6.69	353	355	**191**–173–179	173–171–**126**
**3**	4-Caffeoyl-quinic acid	7.06	353	355	**173**–179–191	**155**
**4**	5-Caffeoyl-quinic acid	7.70	353	355	**191**	173–171–**126**
**20b**	Catechin/Epicatechin	10.38	289	291	271–**245**–205–179	227–**203**–161
**21**	Procyanidin B	11.22	577	579	**425**–407–289	**407**–273
**22**	Isomangiferin	11.63	421	423	**301**–331	273–**258**
**5**	Mangiferin	12.42	421	423	**301**–331–403	273–**258**
**24**	Procyanidin trimer A-type	16.19	863	865	**711**–573–451–411	**693**
**25**	Procyanidin tetramer B-type ^1^	17.10	576	-	**500**–491–289–567–559–451	-
**26**	Apigenin 6,8-di-C-glucoside	17.91	593	-	**473**–503–383–353	383–**353**
**27**	Procyanidin C	18.95	865	-	739–**695**–577–847–449–425	677–**543**–525–451–405–289
**28**	5-Feruloyl-quinic acid	19.25	367	-	**191**–163	173–171–**127**
**29**	Rutin glycoside	19.89	771	773	753–609–**591**–301–300–271	547–445–409–367–**355**–301
**30**	Quercetin sophoroside	20.70	625	627	505–463–445–**301**	**271**–255–179
**31a**	Cinchonain I isomer	21.75	451	-	**341**	231–**217**
**32**	Kaempferol triglycoside	22.41	755	-	**575**–285	393–**339**
**6**	Rutin	23.36	609	611	**301**	271–**179**–151
**33**	3,4-Dicaffeoyl-quinic acid	24.00	515	-	**353**–335	191–179–**173**
**34**	3,5-Dicaffeoyl-quinic acid	24.62	515	-	**353**	**191**–179–173
**35**	Kaempferol-3-O-rhamnoglucoside	25.50	593	-	**285**	267–**257**–241
**36**	4,5-Dicaffeoyl-quinic acid	26.44	515	-	**353**	191–179–**173**
**37**	6-O-(*p*-hydroxybenzoyl)mangiferin	27.10	541	-	**331**–301	**313**–301–271–259
**31b**	Cinchonain I isomer	28.52	451	-	**341**	231–**217**
**38**	Trigonelline ^2^	1.11	-	138	**121**	-
**39**	Caffeine ^2^	20.69	-	195	**138**	**121**

^1^ Identified as doubly deprotonated molecules ([M−2H]^2−^). ^2^ Spectra and fragments acquired in ESI positive mode.

**Table 4:** Concentration of major bioactive compounds found in *C. arabica* L. leaves extracts.

No.	Compound	g/kg DW	%RSD
**1**	3-CGA	1.28 ± 0.12	9.2
**3**	4-CGA	0.89 ± 0.07	8.0
**4**	5-CGA	16.27 ± 1.66	10.2
**34**	3,5-dCQA	0.58 ± 0.02	3.4
**33**	3,4-dCQA	0.63 ± 0.05	7.6
**36**	4,5-dCQA	0.91 ± 0.05	5.3
**5**	Mangiferin	4.43 ± 0.14	3.3
**22**	Isomangiferin	0.52 ± 0.03	5.8
**39**	Caffeine	7.94 ± 0.42	5.3
**38**	Trigonelline	4.47 ± 0.13	2.9

## Data Availability

The data presented in this study are available on request from the corresponding author.

## References

[1] Campa C., Mondolot L., Rakotondravao A., Bidel L.P.R., Gargadennec A., Couturon E., La Fisca P., Rakotomalala J.-J., Jay-Allemand C., Davis A.P. (2012). A Survey of Mangiferin and Hydroxycinnamic Acid Ester Accumulation in Coffee (Coffea) Leaves: Biological Implications and Uses. Ann. Bot..

[2] Chen X. (2019). A Review on Coffee Leaves: Phytochemicals, Bioactivities and Applications. Crit. Rev. Food Sci. Nutr..

[3] Ngamsuk S., Huang T.C., Hsu J.L. (2019). Determination of Phenolic Compounds, Procyanidins, and Antioxidant Activity in Processed *Coffea arabica* L. Leaves. Foods.

[4] Alañón M.E., Palomo I., Rodríguez L., Fuentes E., Arráez-Román D., Segura-Carretero A. (2019). Antiplatelet Activity of Natural Bioactive Extracts from Mango (*Mangifera Indica*, L.) and its By-Products. Antioxidants.

[5] Bonechi C., Donati A., Tamasi G., Leone G., Consumi M., Rossi C., Lamponi S., Magnani A. (2018). Protective Effect of Quercetin and Rutin Encapsulated Liposomes on Induced Oxidative Stress. Biophys. Chem..

[6] Tamasi G., Pardini A., Croce R., Consumi M., Leone G., Bonechi C., Rossi C., Magnani A. (2021). Combined Experimental and Multivariate Model Approaches for Glycoalkaloid Quantification in Tomatoes. Molecules.

[7] Monteiro Â., Colomban S., Azinheira H.G., Guerra-Guimarães L., Do Céu Silva M., Navarini L., Resmini M. (2019). Dietary Antioxidants in Coffee Leaves: Impact of Botanical Origin and Maturity on Chlorogenic Acids and Xanthones. Antioxidants.

[8] Pardini A., Consumi M., Leone G., Bonechi C., Tamasi G., Sangiorgio P., Verardi A., Rossi C., Magnani A. (2021). Effect of Different Post-Harvest Storage Conditions and Heat Treatment on Tomatine Content in Commercial Varieties of Green Tomatoes. J. Food Comp. Anal..

[9] Perrone D., Donangelo R., Donangelo C.M., Farah A. (2010). Modeling Weight Loss and Chlorogenic Acids Content in Coffee during Roasting. J. Agric. Food Chem..

[10] Esquivel P., Viñas M., Steingass C.B., Gruschwitz M., Guevara E., Carle R., Schweiggert R.M., Jiménez V.M. (2020). Coffee (*Coffea arabica* L.) by-Products as a Source of Carotenoids and Phenolic Compounds—Evaluation of Varieties with Different Peel Color. Front. Sustain. Food Syst..

[11] Dar A., Faizi S., Naqvi S., Roome T., Zikr-ur-Rehman S., Ali M., Firdous S., Moin S.T. (2005). Analgesic and Antioxidant Activity of Mangiferin and its Derivatives: The Structure Activity Relationship. Biol. Pharm. Bul..

[12] Segheto L., Santos B.C.S., Werneck A.F.L., Vilela F.M.P., de Sousa O.V., Rodarte M.P. (2018). Antioxidant Extracts of Coffee Leaves and its Active Ingredient 5-Caffeoylquinic Acid Reduce Chemically-Induced Inflammation in Mice. Ind. Crops Prod..

[13] Chen X.M., Ma Z., Kitts D.D. (2018). Effects of Processing Method and Age of Leaves on Phytochemical Profiles and Bioactivity of Coffee Leaves. Food Chem..

[14] Boros K., Jedlinszki N., Csupor D. (2016). Theanine and Caffeine Content of Infusions Prepared from Commercial Tea Samples. Pharmacogn. Mag..

[15] Choung M.G., Hwang Y.S., Lee M.S., Lee J., Kang S.T., Jun T.H. (2014). Comparison of Extraction and Isolation Efficiency of Catechins and Caffeine from Green Tea Leaves Using Different Solvent Systems. Int. J. Food Sci. Technol..

[16] Tamasi G., Baratto M.C., Bonechi C., Byelyakova A., Pardini A., Donati A., Leone G., Consumi M., Lamponi S., Magnani A. (2019). Chemical characterization and antioxidant properties of products and by-products from *Olea europaea* L.. Food Sci. Nutr..

[17] Tamasi G., Pardini A., Bonechi C., Donati A., Pessina F., Marcolongo P., Gamberucci A., Leone G., Consumi M., Magnani A. (2019). Characterization of Nutraceutical Components in Tomatoes Pulp, Skin and Locular Gel. Eur. Food Res. Technol..

[18] Tamasi G., Bonechi C., Leone G., Andreassi M., Consumi M., Sangiorgio P., Verardi A., Rossi C., Magnani A. (2021). Varietal and Geographical Origin Characterization of Peaches and Nectarines by Combining Analytical Techniques and Statistical Approach. Molecules.

[19] Zielińska A., Siudem P., Paradowska K., Gralec M., Kaźmierski S., Wawer I. (2020). Aronia Melanocarpa Fruits as a Rich Dietary Source of Chlorogenic Acids and Anthocyanins: 1H-NMR, HPLC-DAD, and Chemometric Studies. Molecules.

[20] Gogna N., Hamid N., Dorai K. (2015). Metabolomic Profiling of the Phytomedicinal Constituents of *Carica papaya* L. Leaves and Seeds by 1H NMR Spectroscopy and Multivariate Statistical Analysis. J. Pharm. Biomed. Anal..

[21] Ulrich E.L., Akutsu H., Doreleijers J.F., Harano Y., Ioannidis Y.E., Lin J., Livny M., Mading S., Maziuk D., Miller Z. (2007). BioMagResBank. Nucleic Acids Res..

[22] Wishart D.S., Guo A., Oler E., Wang F., Anjum A., Peters H., Dizon R., Sayeeda Z., Tian S., Lee B.L. (2022). HMDB 5.0: The Human Metabolome Database for 2022. Nucleic Acids Res..

[23] Malherbe C.J., Willenburg E., De Beer D., Bonnet S.L., Van der Westhuizen J.H., Joubert E. (2014). Iriflophenone-3-C-Glucoside from Cyclopia Genistoides: Isolation and Quantitative Comparison of Antioxidant Capacity with Mangiferin and Isomangiferin Using on-Line HPLC Antioxidant Assays. J. Chromatogr. B Anal. Technol. Biomed. Life Sci..

[24] Barreto J.A.C.B., Revisan M.A.T.S.T., Ull W.I.E.H., Piegelhalder B.E.S., Wen R.O.W.O. (2008). Characterization and Quantitation of Polyphenolic Compounds in Bark, Kernel, Leaves, and Peel of Mango (*Mangifera indica* L.). J. Agric. Food Chem..

[25] Clifford M.N., Johnston K.L., Knight S., Kuhnert N. (2003). Hierarchical Scheme for LC-MS n Identification of Chlorogenic Acids. J. Agric. Food Chem..

[26] Clifford M.N., Kirkpatrick J., Kuhnert N., Roozendaal H., Salgado P.R. (2008). LC-MSn Analysis of the Cis Isomers of Chlorogenic Acids. Food Chem..

[27] Clifford M.N., Knight S., Kuhnert N. (2005). Discriminating between the Six Isomers of Dicaffeoylquinic Acid by LC-MSn. J. Agric. Food Chem..

[28] Clifford M.N., Marks S., Knight S., Kuhnert N. (2006). Characterization by LC-MS n of Four New Classes of p-Coumaric Acid-Containing Diacyl Chlorogenic Acids in Green Coffee Beans. J. Agric. Food Chem..

[29] Jaiswal R., Patras M.A., Eravuchira P.J., Kuhnert N. (2010). Profile and Characterization of the Chlorogenic Acids in Green Robusta Coffee Beans by LC-MSn: Identification of Seven New Classes of Compounds. J. Agric. Food Chem..

[30] Panche A.N., Diwan A.D., Chandra S.R. (2016). Flavonoids: An Overview. J. Nutr. Sci..

[31] Kazuno S., Yanagida M., Shindo N., Murayama K. (2005). Mass Spectrometric Identification and Quantification of Glycosyl Flavonoids, Including Dihydrochalcones with Neutral Loss Scan Mode. Anal. Biochem..

[32] Salerno R., Casale F., Calandruccio C., Procopio A. (2016). Characterization of Flavonoids in *Citrus bergamia* (Bergamot) Polyphenolic Fraction by Liquid Chromatography–High Resolution Mass Spectrometry (LC/HRMS). PharmaNutrition.

[33] de Almeida R.F., Trevisan M.T.S., Thomaziello R.A., Breuer A., Klika K.D., Ulrich C.M., Owen R.W. (2019). Nutraceutical Compounds: Echinoids, Flavonoids, Xanthones and Caffeine Identified and Quantitated in the Leaves of *Coffea arabica* Trees from Three Regions of Brazil. Food Res. Int..

[34] Stöggl W.M., Huck C.W., Bonn G.K. (2004). Structural Elucidation of Catechin and Epicatechin in Sorrel Leaf Extracts Using Liquid-Chromatography Coupled to Diode Array-, Fluorescence-, and Mass Spectrometric Detection. J. Sep. Sci..

[35] Wang Y., Berhow M.A., Black M., Jeffery E.H. (2020). A Comparison of the Absorption and Metabolism of the Major Quercetin in Brassica, Quercetin-3-O-Sophoroside, to That of Quercetin Aglycone, in Rats. Food Chem..

[36] Justesen U., Arrigoni E. (2001). Electrospray Ionisation Mass Spectrometric Study of Degradation Products of Quercetin, Quercetin-3-Glucoside and Quercetin-3-Rhamnoglucoside, Produced by in Vitro Fermentation with Human Faecal Flora. Rapid Commun. Mass Spectrom..

[37] Khallouki F., Ricarte I., Breuer A., Owen R.W. (2018). Characterization of Phenolic Compounds in Mature Moroccan Medjool Date Palm Fruits (*Phoenix dactylifera*) by HPLC-DAD-ESI-MS. J. Food Comp. Anal..

[38] Angeloni S., Navarini L., Khamitova G., Sagratini G., Vittori S., Caprioli G. (2020). Quantification of Lignans in 30 Ground Coffee Samples and Evaluation of Theirs Extraction Yield in Espresso Coffee by HPLC-MS/MS Triple Quadrupole. Int. J. Food Sci. Nutr..

[39] Resende F.O., Rodrigues-Filho E., Luftmann H., Petereit F., De Mello J.C.P. (2011). Phenylpropanoid Substituted Flavan-3-Ols from Trichilia Catigua and Their in Vitro Antioxidative Activity. J. Braz. Chem. Soc..

[40] De Marchi F., De Rosso M., Flamini R. (2022). Coupling between High-Resolution Mass Spectrometry and Focalized Data-Analysis Methods Provides the Identification of New Putative Glycosidic Non-Anthocyanic Flavonoids in Grape. Metabolomics.

[41] Dantas C.A.G., Abreu L.S., da Cunha H.N., Veloso C.A.G., Souto A.L., de Fátima Agra M., de Oliveira Costa V.C., da Silva M.S., Tavares J.F. (2021). Dereplication of Phenolic Derivatives of Three Erythroxylum Species Using Liquid Chromatography Coupled with ESI-MSn and HRESIMS. Phytochem. Anal..

[42] Sun W., Miller J.M. (2003). Tandem Mass Spectrometry of the B-Type Procyanidins in Wine and B-Type Dehydrodicatechins in an Autoxidation Mixture of (+)-Catechin and (−)-Epicatechin. J. Mass Spectrom..

[43] Rockenbach I.I., Jungfer E., Ritter C., Santiago-Schübel B., Thiele B., Fett R., Galensa R. (2012). Characterization of Flavan-3-Ols in Seeds of Grape Pomace by CE, HPLC-DAD-MS^n^ and LC-ESI-FTICR-MS. Food Res. Int..

[44] Jungfer E., Zimmermann B.F., Ruttkat A., Galensa R. (2012). Comparing Procyanidins in Selected Vaccinium Species by UHPLC-MS 2 with Regard to Authenticity and Health Effects. J. Agric. Food Chem..

[45] Martini S., Conte A., Tagliazucchi D. (2017). Phenolic Compounds Profile and Antioxidant Properties of Six Sweet Cherry (*Prunus avium*) Cultivars. Food Res. Int..

[46] Rue E.A., Rush M.D., van Breemen R.B. (2018). Procyanidins: A Comprehensive Review Encompassing Structure Elucidation via Mass Spectrometry. Phytochem. Rev..

[47] He B., Li Q., Jia Y., Zhao L., Xiao F., Lv C., Xu H., Chen X., Bi K. (2012). A UFLC-MS/MS Method for Simultaneous Quantitation of Spinosin, Mangiferin and Ferulic Acid in Rat Plasma: Application to a Comparative Pharmacokinetic Study in Normal and Insomnic Rats. J. Mass Spectrom..

[48] Rodríguez-Gómez R., Vanheuverzwjin J., Souard F., Delporte C., Stevigny C., Stoffelen P., De Braekeleer K., Kauffmann J.-M. (2018). Determination of Three Main Chlorogenic Acids in Water Extracts of Coffee Leaves by Liquid Chromatography Coupled to an Electrochemical Detector. Antioxidants.

